# Are There Limitations to Exercise Benefits in Peripheral Arterial Disease?

**DOI:** 10.3389/fcvm.2018.00173

**Published:** 2018-11-27

**Authors:** Madaniah Zakari, Musaad Alsahly, Lauren G. Koch, Steven L. Britton, Laxmansa C. Katwa, Robert M. Lust

**Affiliations:** ^1^Department of Physiology, Brody School of Medicine, East Carolina University, Greenville, NC, United States; ^2^Department of Physiology, College of Medicine, Taibah University, Al-Madinah Al-Munawwarah, Saudi Arabia; ^3^Department of Physiology and Pharmacology, University of Toledo, Toledo, OH, United States; ^4^Departments of Anesthesiology and Molecular and Integrative Physiology, University of Michigan, Ann Arbor, MI, United States

**Keywords:** innate, rats, intrinsic exercise capacity, limits, disease models

## Abstract

Substantial evidence exists indicating that inactivity contributes to the progression of chronic disease, and conversely, that regular physical activity can both prevent the onset of disease as well as delay the progression of existing disease. To that end “exercise as medicine” has been advocated in the broad context as general medical care, but also in the specific context as a therapeutic, to be considered in much the same way as other drugs. As there are non-responders to many medications, there also are non-responders to exercise; individual who participate but do not demonstrate appreciable improvement/benefit. In some settings, the stress induced by exercise may aggravate an underlying condition, rather than attenuate chronic disease. As personalized medicine evolves with ready access to genetic information, so too will the incorporation of exercise in the context of those individual genetics. The focus of this brief review is to distinguish between the inherent capacity to perform, as compared to adaptive response to active exercise training in relation to cardiovascular health and peripheral arterial disease.

## Why exercise?

Exercise is a set of physical activities requiring a coordinated series of homeostatic responses from the body to support increased workloads applied to skeletal muscle. Exercise is a form of stressful stimuli that leads to specific changes in the physiology. When the internal homeostasis is imbalanced by any physical activity, the cellular response to the exercise will limit the internal stress in an adapting effort to maintain normal homeostasis, both in the current exposure, but also in anticipation of future activity exposures. The best example of the integrated effect is the overall impact on the autonomic nervous system, controlling most body systems, and showing a shift toward more parasympathetic resting tone with exposure to regular exercise. In fact, the presence of an active exercise “training effect” most commonly is a decrease in resting heart rate, coupled with an increased oxygen efficiency (1).

Humans engage in exercise for three primary reasons. First, individuals find pleasure in the activity. While that may be true for some, it is not a universal finding. The second reason is that a health benefit has been appreciated, and regardless of the predisposition for exercise, the benefits of exercise in promoting health or limiting disease progression is appreciated. The third reason for exercise is as an adjuvant therapy in the recovery from acute injury. Exercise for rehabilitation is used for individuals to help them recover from current injury or live with chronic medical conditions (1).

## Health benefits of exercise

Studies have revealed that regular exercise has a favorable effect in the promotion of health and treatment of diseases. This reality has led several national associations, including the American Heart Association (AHA), the American Diabetes Association (ADA), and the American College of Sports Medicine (ACSM) to consider/advocate for “exercise as medicine” (2–4). According to Vina et al., exercise leads to wide ranging health benefits, enhancing quality of life in those with disease, and increased longevity in those who are disease free (2). In healthy individuals, exercise reduces the overall risk of death by 20–35% (5, 6). One study showed that middle-aged women who participated in <1 h of exercise per a week, showed a 52% increase in all-cause mortality, including cancer, in comparison with physically active women (7). Other studies have shown that regular exercise could be used to prevent cardiovascular and pulmonary diseases, including coronary heart diseases, chronic obstructive pulmonary disease, hypertension, and intermittent claudication; metabolic syndromes (type 2 diabetes, obesity, and hyperlipidemia); cancer; depression; musculoskeletal diseases including osteoporosis, rheumatoid arthritis, fibromyalgia, chronic fatigue syndrome, and gout (8–12).

These studies indicate that exercise, like other therapeutics, has many of the same properties as a drug when considering the optimal or effective dose response, and suggest the importance of understanding the molecular mechanism of exercise in healthy and diseased conditions. As a result, exercise is now prescribed in dosage form, just like any other drug (2, 13–17), in order to achieve optimal health benefits and optimal therapeutic effects.

There are interesting differences between medical therapy and exercise, at least in the context of a drug. Drugs, for the most part, are used either to treat diseases, or in some cases to prevent them, but rarely for both applications. Statins, for the most part, are prescribed to manage hypercholeresterolemia for individuals with coronary artery disease (CAD). Increasingly, they are also prescribed for asymptomatic individuals without evidence of CAD, and in some cases even without evidence of hypercholesterolemia, as a prophylactic for developing occlusive vascular disease (18). While such dual use is unusual for most drugs (applied to both treatment and prevention), exercise is commonly considered equally efficacious both in the context of disease prevention, as well as disease management (18, 19). Exercise is attractive from that standpoint, because it is widely viewed as a “magic bullet” substituting for multidrug therapies, targeting not only the primary disease, but also many of the co-morbidities associated with any given disease (20). At the same time, most human studies are limited by enrollment, and many are conducted to look for a specific exercise effect on a specific disease presentation, usually often skewed by excluding patients with co-morbidities (19). The 2013 AHA/ACC (American College of Cardiology/American Heart Association) guidelines indicate that heart failure patients have an average of 5–6 co-morbidities, depending on age, and the writing team was clear that all treatment recommendations were limited by that context, even though many of the drugs reviewed for heart failure management are clearly directed toward the co-morbidities (18).

A meta-analysis of clinical trials focused on exercise in patients with intermittent claudication, a common symptom of peripheral arterial disease, showed similar challenges, with a consensus summary that exercise did not improve ABI (ankle-brachial index, an indication of perfusion), nor did it reduce mortality or amputation (19) despite numerous rodent studies that suggested exercise improves all of these end points (20–22). Much like the response to exercise, most diseases are polygenic, and most cardiovascular diseases are complicated by multiple co-morbidities including hypertension, coronary artery disease, hyperlipidemia, chronic kidney disease, diabetes, and chronic obstructive pulmonary disease (COPD) (18, 20). The notion that exercise is a “broad spectrum” therapeutic, simultaneously improving both the disease and any co-morbidities, either as a preventive or as an ongoing treatment is a popular, but likely heroic expectation, given the known variability in training-induced responses (23).

Conversely, inactivity generally has been associated with worse outcomes and accelerated progression of these same diseases (20). It is interesting to note that in the 2013 joint ACC/AHA guidelines for managing heart failure, exercise training has only a very small discussion as a “non-pharmacologic intervention” as an adjuvant therapy for quality of life endpoints: “*exercise training (or regular physical activity) is recommended as safe and effective for patients with heart failure, who are able to participate to improve functional status*” (18). Only 8 of nearly 400 references address the role of exercise in the management of heart failure, and it remains an interesting paradox to note that while there may be increasing willingness to prescribe exercise as a therapeutic option in heart failure patients, the severity of heart failure continues to be defined by the progressive limitations to physical activity.

Vina et al. (2) suggested that an organism takes some time to adapt to any form of exercise to which they are introduced. Therefore, it is necessary to understand the molecular signaling pathways of a particular organism (human being or animal) before prescribing exercise to them appropriately (2). However, some of these pathways may be suppressed or maximally activated already by underlying disease, and without recognizing the interaction, the potential for detrimental effects may increase, as it would with any therapeutic.

## Muscle adaptation to exercise

In as much as spontaneous activity is considered normal, exercise is considered a physiological stimulus for altering gene and protein expression in the skeletal muscle, as opposed to a disease process that might induce similar changes. Yet, despite widespread association between an exercise phenotype and better health, because of the multi-system effects of exercise, establishing the cellular basis for any particular benefit has been difficult (20, 21, 23). One major group of mechanisms includes modulation by reactive oxygen species (ROS). However, the effects of ROS can be multimodal, depending on whether they are considered in the context of acutely modulating a transient response to exercise, or in the context of a system that is chronically overloaded by an exercise stress for which it may not have adequate ROS buffering capacity.

**Figure 1:**
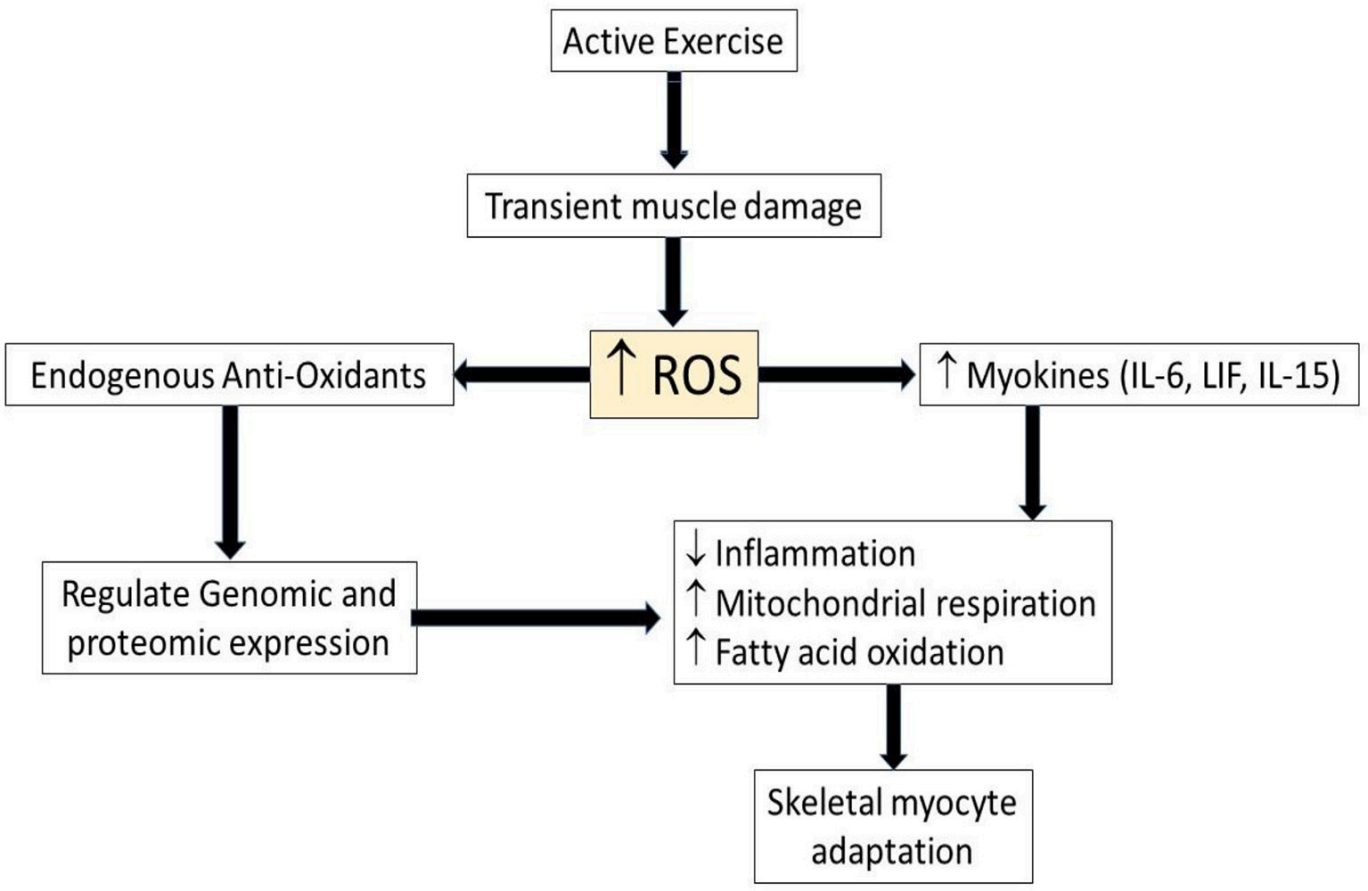
Schematic illustrating role of ROS and myokines in myocyte adaptation during active exercise. Upwards arrows indicate increased level of parameters while downwards arrow indicates decreased level of parameter. ROS, Reactive oxygen species; IL-6, interleukin-6, LIF, leukemia inhibitory factor; IL-15, interleukin-15.

During bouts of exercise, reactive oxygen species (ROS) are produced by the muscle leading to exercise-induced ROS. Although the ROS was associated with cell damage, ROS also is now accepted as a signaling mechanism in muscle adaptation during exercise (Figure 1). For example, exercise-induced ROS stimulate the production of muscle-derived cytokines called “myokines.” These myokines play an important role in prevention of inflammation and the regulation metabolism (24), even though ROS also can be induced by chronic inflammation, and generated by increased metabolic activity. ROS and inflammatory cytokines have paradoxical roles in exercise and disease. Chronic diseases such as type II diabetes and cardiovascular diseases have defects in metabolic function, with recent studies showing the importance of exercise in preventing chronic disease (11). On the other hand, physical inactivity for 2 weeks also caused alteration in the metabolism, such as decreased insulin sensitivity and increased plasma triglycerides (12). Just as the muscle adapts to a low energy level, it also adapts to high energy demand during bouts of exercise, such as increased mitochondrial respiration and fatty oxidation (24). Moreover, exercise increases ROS, which stimulates endogenous antioxidant defense reactions (25) and regulates genomic and proteomic expression (26–28). This suggests that ROS has beneficial effect during muscle adaptation to increased physical activity, but that some of the same effects can be induced by inactivity as well as activity.

**Figure 2:**
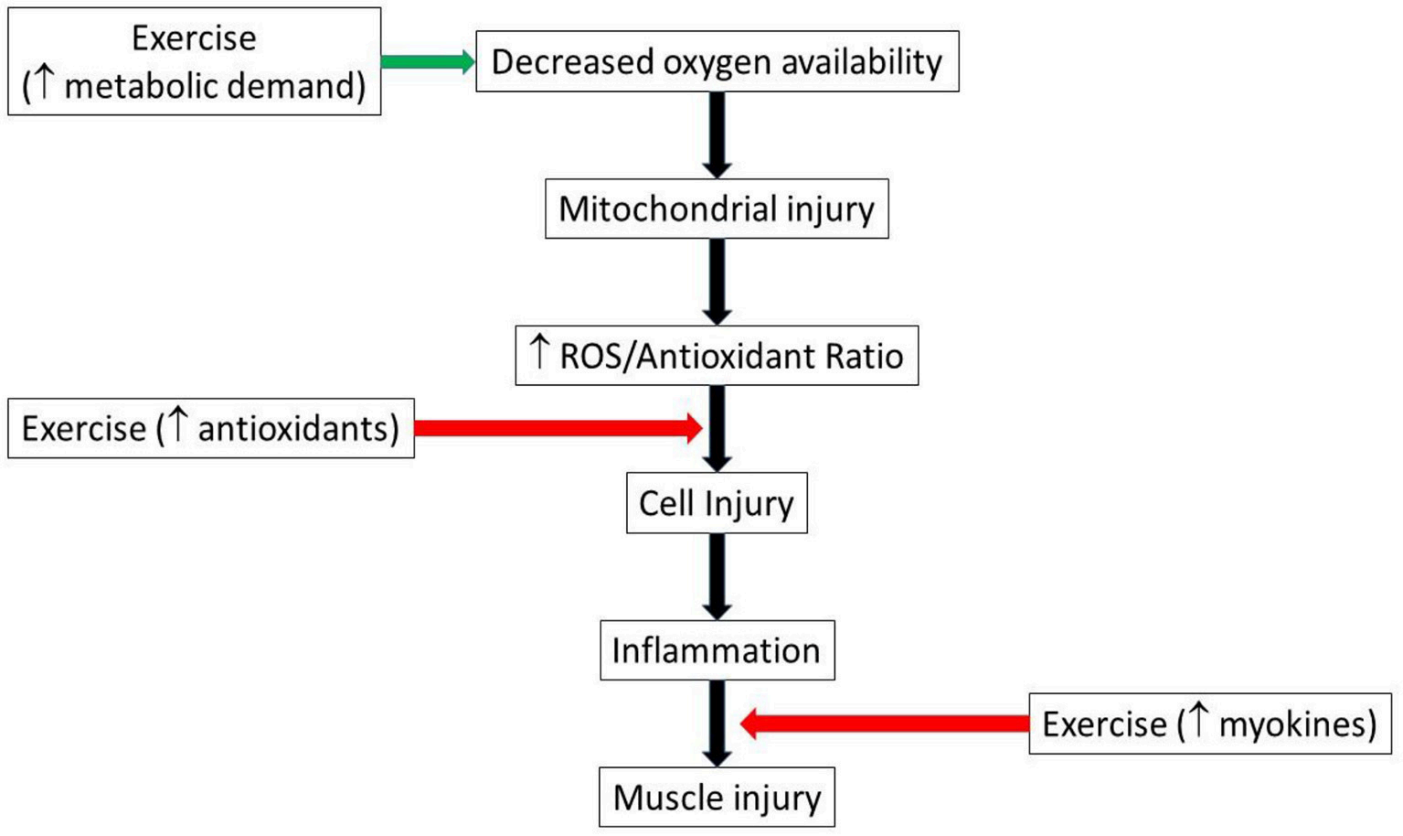
Schematic illustrating possible pathways by which exercise could both aggravate and mitigate muscle injury. Green arrow indicates injury promotion, while red indicates injury limitation pathways. ROS, reactive oxygen species.

Similarly, several cytokines previously associated with cell damage during exercise (24), mirror cytokine expression seen in chronic diseases pathogenesis such as type 2 diabetes (29), cardiovascular disease (30) and neurodegenerative diseases (31). However, exercise-induced myokines play anti-inflammatory roles that scavenge the pro-inflammatory cytokines in chronic diseases (24). Therefore, endogenous antioxidants and myokines are important factors in muscle adaptation to the inflammatory diseases, suggesting a potential benefit of chronic exercise training in the setting of acute ischemic injury (Figure 2).

One example of the beneficial role of ROS during exercise is increased redox-sensitive transcription factors which increase synthesis of peroxisome proliferator-activated receptor gamma coactivator 1-alpha (PGC1α), a transcriptional co-activator of genes involved in mitochondrial respiration and biogenesis (32, 33). PGC1α by itself increased ROS and anti-oxidants through increased mitochondrial respiration (24). PGC1α has been proposed previously to be crucial player in the long-term adaptation to exercise (32).

**Figure 3:**
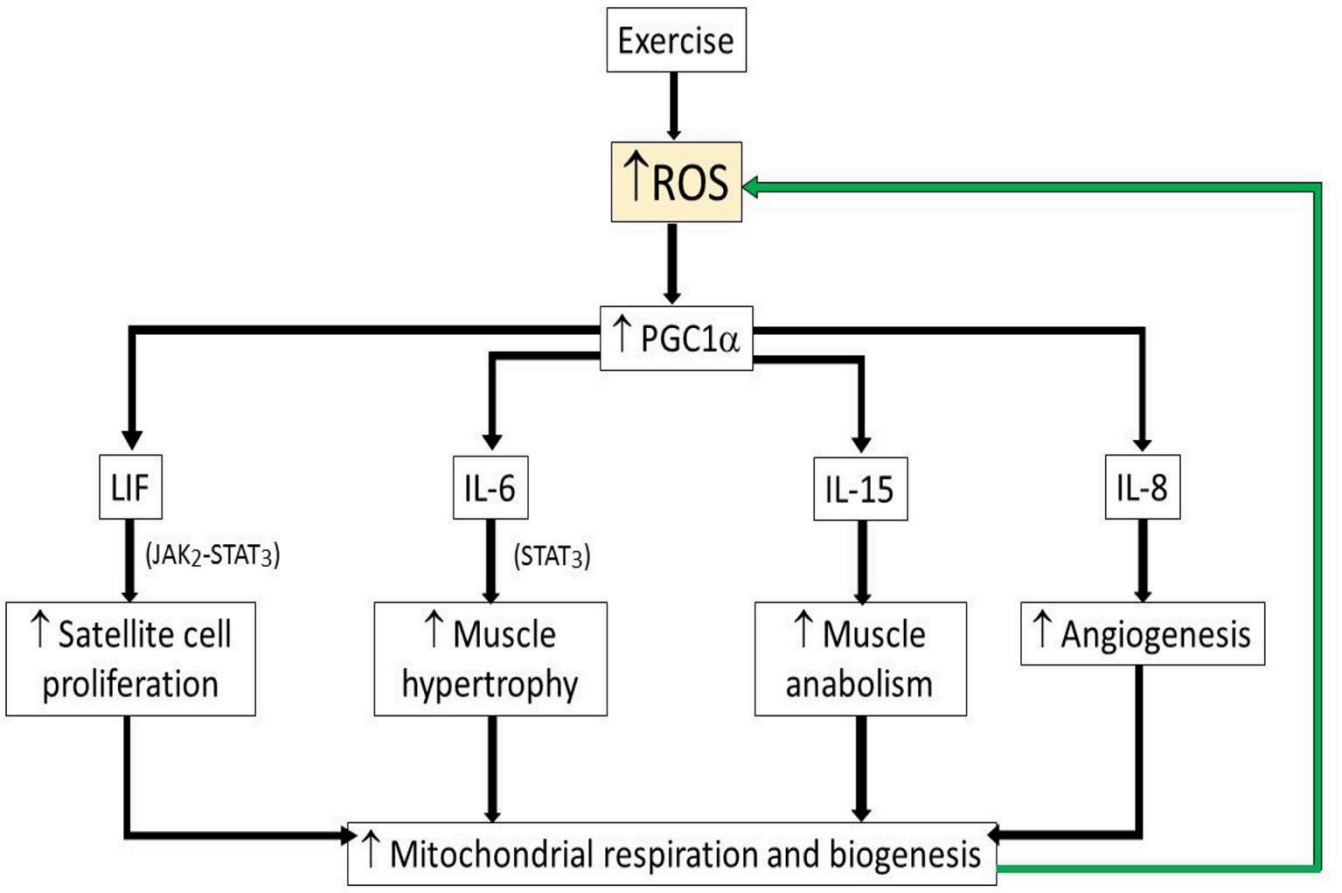
Schematic illustrating beneficial role of ROS during exercise. Upwards arrow indicates increased levels of parameter. Green arrow indicates positive feedback activation. ROS, reactive oxygen species; PGC1α, peroxisome proliferator-activated receptor gamma coactivator 1-alpha; LIF, leukemia inhibitory factor; LI-6, interleukin-6; LI-15, interleukin-15; IL-8, interleukin-8.

On the other hand, ROS also induces myokines such as interleukin-6 (IL-6), leukemia inhibitory factor (LIF), and interleukin-15 (IL-15), which are highly expressed in skeletal muscle after bouts of strength exercise (24). IL-6 was suggested to promote muscle hypertrophy through STAT3, signal transducer and activator of transcription 3 signaling (34) while LIF, a myokine of IL-6 superfamily member, has been shown to increase satellite cell proliferation through the Janus kinase-2/signal transducer and activator of transcription-3 (JAK2-STAT3) signaling pathway (35). IL-15, is a myokine that has been reported to also be an anabolic factor and with increased expressed in skeletal muscle after strength training (36) (Figure 3). These findings indicate that the myokines, which induced during strength training are crucial for muscle adaptation after exercise. While associations of ROS/anti-oxidants also have been the focus of a number of diet based health interventions, it remains that most large clinical studies of anti-oxidants have not demonstrated major health benefits (37, 38), suggesting that moderating ROS cannot explain the health benefits of exercise.

## Other signaling pathway controlled by exercise in the skeletal muscle

Exercise has a curious dichotomy in that many of the signaling pathways associated with beneficial effects of exercise also are associated with harmful effects in many disease processes. As an example, during acute exercise, many effects are activated via adrenergic signaling whose cardiac effects are modulated primarily via the beta-1 adrenergic receptors, while at the same time, many of the medical therapeutics for cardiac diseases include beta blocking drugs as a cornerstone of treatment (18). It remains poorly understood how the cell can differentiate between stimuli to a common pathway, but to either physiologic or pathologic outcomes.

Sakamoto et al. (39) and Ji et al. (40) reported that the cell physiology changes that occur during exercise rely on several stimuli, including: shifts in the adenosine triphosphate: adenosine diphosphate (ATP:ADP) ratio, changes in the concentration of metabolites, alterations in the Ca^2+^ concentration and pH in the cells, and activation of ROS signaling pathway, as mentioned above. Table 1 lists these stimuli. Exercise reportedly activates mitogen-activated protein kinase (MAPK) signaling, including the JNK and extracellular-signal-regulated kinase1/2 (ERK1/2) pathways (41) and p38 MAPK (42). Exercise also activates the adenosine monophosphate-activated protein kinase (AMPK), Akt/PKB (Protein Kinase B) and the p70S6 kinase (39). Intracellular Ca^2+^ levels required for contraction also regulate a host of other intracellular proteins, such as calmodulin kinase, calcineurin and protein kinase C (PKC), which, in turn, mediate intracellular signal transduction (43). As it is shown above that low-to-moderate degrees of reactive oxygen species (ROS) help in controlling the expression of genes, modulating the production of force in the skeletal muscle, and regulating cellular signaling pathways. Angiogenesis also is another important adaptation in skeletal muscle after a period of endurance exercise (44). Interleukin-8 (IL-8) was found to be expressed in skeletal muscle after endurance exercise (45), but IL-8 also has been reported as an angiogenic cytokine in cancer (46). While angiogenesis may be protective in vascular-occlusive diseases, it facilitates progression of tumorogenesis, and it is not at all clear how the cell distinguishes between different stimuli driving the same outcome.

**Table 1:** Lists of the skeletal myocyte physiological stimuli during exercise.

• Changes in ATP:ADP ratio.
• Changes in the metabolite concentrations.
• Changes in intracellular Ca^+2^ concentration.
• Changes in pH.
• Changes in REDOX state.
• Changes in ROS signaling pathway activation.

*Adenosine triphosphate to adenosine diphosphate ratio (ATP:ADP); cationic calcium ion (Ca^+2^); indicator of acidity (pH); relative reduction to oxidative state (REDOX state); reactive oxygen species (ROS)*.

## The dosage of exercise

Medication dosage is critical in clinical medicine, and all drugs need data on their safety and efficacy (47). Vina et al. have reported that every organism needs a minimum amount of exercise, below which there is a detriment to health (2), suggesting a threshold effect. Above the minimum threshold, the advantages accumulating from physical activities increase in proportion to intensity/amount of physical activities. Beyond a particular level, however, a second threshold appears where the negative impacts of exercise tend to offset the advantages. Unlike medical drugs, the minimal dose, maximum safe dose and dose response of exercise vary greatly from one organism to another, eliciting a continuing debate on how much, how often, what type, how lengthy, and with what intensity exercise should be observed (47). The challenges arise because of differing methods in measuring exercise intensity, differing classification of dose schemes (47) and differing phenotypes within the same or similar species. For example, Warburton et al. (48), advocates that the intensity levels of physical training should be measured based on oxygen consumption (VO_2_) or heart rate. Yet the actual readouts for the exercise are behaviorally based, and may or may not correlate with the underlying variables of interest (19). For instance, moderate-intensity exercise would occur when both heart and respiratory rates are increased without interference with the ability to talk, as might be seen in brisk walking with speed of 3.0 mph (80.4 m/min) or what would be equal to about 100 steps/min. However, vigorous-intensity exercise is when the heart and respiratory rates are increased but there is difficulty with maintaining conversation, as in running.

In order to determine the minimal effective dose of exercise for each individual, the exercise dose should be handled clinically as any other pharmacological dosing. One would begin using the minimal effective dose of a drug, and if the patient does not respond effectively, one would start titrating the dose upwards toward the maximal effective dose. Similarly, exercise should be started at minimal effort and titrated toward the maximal effort the individual can tolerate, depend on the current condition (47). While there are some considerations for underlying disease, in general, the recommendations for exercise are fairly uniform (49, 50), and do not account for individual differences in minimum or maximally effective doses, which inevitably means that dosing errors will occur (51).

A recommended exercise regimen for all healthy young to middle-age adults (presuming asymptomatic equates with disease free) might be 300 min or more of moderate-intensity aerobic exercise per week, or 150 min or greater of vigorous-intensity aerobic exercise per week (49, 50). Weekly totals suggest that there is no difference in dosing frequency on outcomes (e.g., daily vs. biweekly), and suggests that the (total duration) X (dose) equation is a constant, which is not at all clear.

Further caution is required for adjusting the exercise dose when prescribed for specific populations such as elderly, children, pregnant women, and patients with comorbidities (48). For example, elderly men over 60 may not require vigorous exercise to prevent cardiovascular risks, and instead may significantly decrease mortality rate with regular low level exercise (2). For type 2 diabetic patients, they might benefit initially from the training levels that recommended for healthy adults and gradually moving up toward the recommended doses for specific condition of each individual (50). For heart failure patients, a low to moderate intensity “60–80% VO_2_,” depends on the patients previous exercise status and disease condition, with 10–15 min warm-up period, 30 min duration, and 3–5 times per week as the optimal training frequency [for more review about exercise and heart failure see AHA recommendation in the circulation (52). Overall, according to Wen et al. (53), 15 min per day or 90 min per week of moderate-intensity physical activity is valuable both to an individual's health and life expectancy, and even for persons with high cardiovascular risks (47). While these guidelines suggest a minimum threshold for benefit, they also imply a significant risk for harm if the dose is exceeded, depending on the specific condition.

As exercise by itself is a stimulus and a burden that requires several cellular interactions through multiple signaling pathways, exposing the severely ill person to this stimulus might be harmful, especially if the illness also affects similar pathways or cells that are utilized in the response to exercise.

## Intrinsic vs. active exercise

Much of what is understood about the relationship between exercise and health is based on the responses to active exercise, yet twin and family studies supported the heritability of exercise capacity. Based on twin studies, Klissouras estimated that the variability in aerobic running capacity was 93.4% genetically determined in unconditioned humans (54). Later, molecular techniques were used to determine gene markers for endurance capacity, and subsequent studies showed that variations in mitochondrial DNA affected not only baseline oxygen consumption (VO_2_ max), but also influenced the capacity for increasing VO_2_ max with exercise training. Using 415 sibling pairs from the HERITAGE family study, Bouchard and colleagues evaluated VO_2_ max during a sedentary phase and after a training phase (55). The VO_2_ response was adjusted for the effects of age, sex, body mass, fat mass, and fat free mass. After the training phase, VO_2_ was adjusted for age and baseline VO_2_. Using a single point linkage procedure, several specific chromosomal regions were identified to be linked to the VO_2_ max outcome (55).

By 2005, 17 mitochondrial genes from 165 autosomal gene entries had been identified to play a role in observed fitness and performance phenotypes (56). Genes involved in aerobic metabolism are down regulated in metabolic disease states and may be linked to disease pathogenesis. Markers that identify an individual with affirmative traits for physical capacity are also related to gene markers that influence body composition and obesity status.

A series of studies conducted by Bouchard and Tremblay assessed the evidence for genotypes associated with overfeeding or negative energy balance and their interaction effects in the changes in body composition, body weight, and body fat distribution (57). Utilizing twin pairs design, the researchers showed that exposure to overfeeding or negative calorie balance were much more alike in the twins than those who were not genetically related, and that among those unrelated, there was a heterogeneous response to these conditions (57).

The study by Bouchard & Tremblay utilized exercise as a means to achieve negative calorie balance. Studies of gene markers for exercise intolerance also have been studied. Rankinen et al. have summarized all of the genes encoded by nuclear or mitochondrial DNA in which mutations have been reported in patients with exercise intolerance (56). Genes receiving several annotations include CPT2 (carnitine palmitoyl transferase II), at the 1p32 chomosomal location, PGAM2 (phosphoglycerate mutase 2), located at 7p13.12, and PFKM (phosphofructokinase—muscle), located at 12q13.3.

Utilizing the Quebec Family Study, Jacobson et. al. studied genes influencing resting metabolic rate (RMR) and respiratory quotient (RQ), and were targeted because of the relationship between oxidative capacity and either energy balance or substrate oxidation (58). They determined a linkage to RMR on chromosomes 3q25.1 (lod_2.74), 1q21.2 (2.74), and 22q12.3 (1.33). Linkages to RQ were found on chromosomes 12q13 (1.65) and 14q22 (1.83). This data is important because the chromosomal linkages to RMR were previously associated with metabolic syndrome, and were affirmed again in this study, which clarifies a genetic relationship between those factors that influence metabolic rate and metabolic disease.

It has been difficult to determine the role that aerobic capacity plays on the progression of disease states, since it is nearly impossible to control for the confounding genetic and environmental influences that influence outcomes. In order to improve the ability to look at the role of muscle oxidative capacity in disease progression, Koch and Britton developed a rat model with contrasting differences in running capacity phenotypes (59).

Using a genetically heterogeneous outbred stock of N:NIH rats, artificial selection for intrinsic aerobic endurance capacity was initiated with a founder population of 80 male and 88 female rats. Genetic variance among the population was maximized by not selecting among brothers and sisters. At 10 weeks of age, a protocol for estimating aerobic running capacity was used (60). The 13 rats with the highest running capacity (HCR) and the 13 with the lowest capacity (LCR) were selected from each sex and randomly paired for mating. At 10 weeks of age, the offspring again were introduced to the treadmill protocol and the selection of the high and low capacity groups was repeated. This method continued over several generations. Genetic heritability of the phenotype for aerobic capacity was clearly demonstrated by a difference in running capacity of 347% between groups at generation 11. Treadmill running capacity decreased by an average of 16 min per generation for the LCRs, and increased by an average of 41 min per generation in the HCRs in response to the selection. Presently, the colony is in generation 41, with a stable difference in aerobic capacity between the phenotypes of at least 1000%. Since the inception of the colony about 15 years ago, about 200 papers have been published using the model, and the results have been reviewed recently (61–64).

Generally, the HCR animals have been characterized as “disease resistant” while the LCR's have come to be characterized as “disease prone” generally due to their predisposition for traits commonly associated with human disease, such as metabolic syndrome, susceptibility to weight gain on high fat diets, reduced endothelial function, and impaired energetics (61–65). Interestingly, the intrinsic capacity for exercise did not predict the ability to respond to an active training protocol (66), and the genetics associated with the induced response to training are distinct and separate from the intrinsic capacity for aerobic exercise. Nonetheless, recent experiments have suggested that active exercise might “rescue” the LCR phenotype in a model of post-operative neuro-inflammation (67).

As mentioned at the outset, humans engage in exercise for several reasons. First, individuals find pleasure in the activity. While that may be true for some, it is not a universal finding. The second reason is that a health benefit has been appreciated, and regardless of the predisposition for exercise, the benefits of exercise in promoting health or limiting disease progression is appreciated. Humans might engage in exercise they don't particularly find rewarding (missing reason one) because they have assigned a higher value to exercise in overall health. While humans will sometimes be motivated by a higher purpose to engage in activity they would otherwise be inclined to avoid, rodents generally do not. In the Koch and Britton animals, the selection is based on maximum performance in forced treadmill running, which they referred to as intrinsic running capacity. There likely is a behavioral component to that performance. For example, we have reported that HCR animals persevered nearly twice as long in a forced swim test (378 ± 18 vs. 189 ± 19 s; *p* < 0.05), but also less likely to explore open arms of an elevated plus maze (378 ± 18 vs. 189 ± 19 s; *p* < 0.05) and higher aggression scores in response to handling (6.1 ± 0.9 vs. 1.7 ± 0.4; *p* < 0.05). Together, these would indicate that along with aerobic endurance running, HCRs also selected for perseverance in futile activity, anxiety, and aggressiveness (68). Still, it seems clear from studies of isolated tissues that the differences are between the LCR and HCR in the Koch Britton model are not driven primarily by the behavioral component (61–64).

Booth and his colleagues approached the exercise capacity issue using a similar phenotypic selection approach, but based primarily on the behavioral component, suggesting that the primary driving factor in exercise capacity was motivational (69). In this case, selection was based not on forced treadmill running, but on voluntary wheel running. In evaluating those animals a strong relationship was identified between dopaminergic receptor levels, and increased capacity for voluntary wheel running which could be significantly attenuated by dopamine receptor antagonism (69). The model has been highly valuable in defining the neural pathways associated with the intrinsic reward pathways associated with increased voluntary wheel running. The challenge is that voluntary wheel running declines rapidly with age in rats, starting as early as 8 weeks (20), to such a degree that running wheels are not considered a routine part of normal husbandry enrichment for rats, while they are for mice. Still, the findings between the Koch and Britton strains, and the Booth strains are complementary, and likely each represent a component of the human overall response to exercise. In either case, reduced capacity is associated with poor outcomes, and increased capacity is associated with disease resistance.

The third reason for exercise is as an adjuvant therapy in the recovery from acute injury. Exercise for rehabilitation is used for individuals to help them recover from current injury or live with chronic medical conditions (1). In general, this would be applied in settings after an injury, recently painful, and for which a motivational element most surely plays a role. A lack of motivation, coupled with a lack of capacity is a sure recipe for a failed intervention, which is not uncommon with cardiovascular diseases (18, 19). To the extent that damage can operate in the same signaling pathways as exercise, there may be a very fine line between added injury and enhanced recovery.

## Could exercise be harmful?

In certain circumstances, exercise could turn out to be harmful instead of being advantageous, as one might have expected/hoped. In this circumstance, exercise either hurts a person's health or fails to bring the expected benefits. While it might be suggested that failure to improve is not the same as harm, and as long as there is no overt harm demonstrated, then why not prescribe for all? Yet, there are few medications that are prescribed in general, without specific intention for a designated benefit. We used the HCR/LCR model to explore some of those issues.

## Is high inborn aerobic capacity alone a protective internal factor for cardiovascular disease?

Maximal oxygen uptake (Vo_2max_) is one of the strong predictive markers for mortality and morbidity (70, 71), but that is largely based on induced responses, and the cardio-protective effect of high intrinsic Vo_2max_ remains unclear (72). We reported that LCR and HCR rats each subjected to acute regional ischemia reperfusion injury *in vivo* (30 min ligation followed by 2 hours of reperfusion), produced no difference in the infarct size between HCRs and LCRs (73). However, when the ischemic time was reduced to 15 min, the HCRs had a significantly smaller infarction, suggesting two things: there is likely a level of injury for which no level of exercise is protective, and once the protective level is surpassed, the level of injury expansion is accelerated (the difference in injury between 15 and 30 min of ischemia was greater in HCRs than LCRs). These data might suggest that it is the inducible exercise capacity profile of genes that is more critical to tolerance for acute injury, while it is the inherent exercise gene pool that provides elements of resistance or susceptibility to chronic disease. In similar experiments, Høydal et al. also demonstrated that the impacts of myocardial infarction in HCR rats were similar or even more prevalent on cardiomyocyte and cardiac—contractile function, and did not reinforce a cardio-protective impact of high inborn aerobic capacity. The authors also suggested that the cardio-protection associated with high aerobic capacity or VO_2max_ might only depend on cardiac preconditioning and acquired aerobic capacity, and not on inborn characteristics alone (72).

## Does diabetes attenuate the benefits of exercise in PAD patients and an animal model of peripheral arterial disease?

Because the field of exercise intervention in cardiovascular diseases is huge, we kept the title of this review is focused on peripheral arterial disease (PAD) only in human and animals to help in improving the transitional understanding between the animal and human. In PAD patients exhibiting intermittent claudication (IC), supervised and well-coordinated exercise is an instrumental medication (75). It is noteworthy, nonetheless, that the majority of PAD patients don't exhibit typical IC symptoms, and there is considerable disagreement in the literature surrounding the impact of exercise on PAD patients (19). In 2016, Lyu et al. (75) conducted a meta-analysis on studies up to 2014, in patients with PAD with and without intermittent claudication using *Pubmed, Ovid Embase*, and *The Cochrane Library* as the sources for relevant studies. They select randomized clinical controlled trials to compare between intensive walking exercise with common advice such as ‘go home and walk’ (75). This study involved 18 trials with an aggregate of 1200 PAD patients. The period of the exercise program was 12 weeks and above (76). The assessment of walking was reported as maximum walking distance (MWD), pain free walking distance (PFWD), and their times (MWT and PFWT), respectively (75), measured during a graded treadmill test.

The results from the study indicated that regardless of exercise modality and length, steadily intensive walking exercise boosted the walking capability of patients with PAD more than usual care (75). However, diabetes as a comorbidity attenuated most of the gains in the walking performance in PAD patients after the exercise (75). The study did not assess longer term outcomes, but the lack of benefit, when there was one in the control group, could suggest the potential for exercise stress in this setting to exacerbate the underlying disease.

In 2017, another systematic meta-analysis was conducted to further clarify whether supervised exercise therapy is harmful for some patients with intermittent claudication due to underlying comorbidities. In this case, Hageman et al. (77) was more specific and focused on the influence of diabetes mellitus (DM) on walking distance during supervised exercise treatment for PAD with intermittent claudicated patients. The systematic review again included randomized and non-randomized studies that found in *Medline, Embase*, and *PubMed Central* (77). Similar to the previous study by Lyu (75), the assessment of walking was based on MWD, PFWD, and functional walking distance (FWD). The length of exercise therapy was at least 3 months, three times per week. The walking distance was based on treadmill protocol with a speed of 3.2 kph (2 mph) and gradually increasing slope 2% every 2 min to reach maximum level of 10% (78–80). Three studies fit the inclusion criteria, and using these more narrowly defined criteria, the major conclusion was that there was no significance difference between diabetic and non-diabetic groups after the exercise therapy, and no impairment in the walking parameters after exercise in the diabetic group except for one small study (*n* = 60), where there was a borderline significant (*p* = 0.056) impairment of walking parameters after exercise therapy in diabetic patients with intermittent claudication (81). Although both Lyu and Hageman teams both were interest in the effect of diabetes mellitus on the exercise response during treatment of PAD patients, there are some differences between the two studies. Lyu et al. studied large, randomized, controlled clinical trials for PAD patients with and without intermittent claudication, and analyzed diabetic subgroup as an addition data within different variables (75). Hageman et al. reviewed systematically three studies with small samples size and with the main focus on the comparison between the diabetic and non-diabetic groups without further attention to characteristics of PAD (77). The fact that meta-analysis is the primary way to generate sufficiently powered data on interactions between exercise and underlying disease underscores the limits of the current research field to generate interpretable information both on benefit as well as potential harm.

Several individual studies excluded from the Hageman meta-analysis because they were not exclusively supervised only exercise studies, are noteworthy nonetheless. In 1999, Ubels et al. compared the effect of diabetes mellitus on the home-based but not supervised-based exercise training in intermittent claudicated patients and found that diabetic patients responded better from the exercise therapy than non-diabetic group (82). In 2011, a study conducted by Collins et al. on the effect of 6 months home-based exercise treatment on only diabetic patients and found there was no effect of exercise on improving walking parameters of intermittent claudicated patients with diabetes (83). In 2013, McDermott et al. reported that home based exercise therapy for 6 months produced improvements in the six min walking distance in the diabetic and non-diabetic group (84), but failed to compare the changes between the groups. However, the author did not compare outcomes between the diabetic and non-diabetic groups (77).

To further explore these results in an experimental model, we used low (LCR) and high (HCR) intrinsic aerobic capacity rats as genetic model of metabolic syndrome and non-metabolic syndrome (65), respectively, in a hind limb occlusion model of peripheral arterial obstructive disease (PAOD). In addition, in an effort to determine the benefit/harm of active exercise, animals in each group underwent either an exercise regimen prior to ligation but not afterwards, (Pre-Ex, “prehab”), an exercise regimen after ligation, but not before (Post-Ex, “rehab”), or ligation only without exercise before or after. The exercise protocol was 2 weeks in duration, 5 days per week. Ligation was introduced unilaterally in the left femoral artery of all groups, with the right femoral vasculature serving as the internal control in each animal. Two weeks after the ligation, tissues were harvested from the right and left legs for gene and protein expression, muscle morphometry, and vascular histology. We found that without any exercise before or after, the HCR had better vascularity and less inflammation 2 weeks after ligation. In large part, the difference could be attributed to increased resting capillarity before ligation, which with better collateral flow reducing the amount if muscle injury and subsequent inflammation. Exercise before ligation did little to improve the outcome, but the animals tolerated post-operative exercise and showed a greater improvement in recovered muscle mass than was seen in ligation alone. In contrast, when the LCRs were exercised before the ligation there was a greater recovery in the LCRs compared to the HCRs, but when the LCRs were exercised after ligation, the outcome was worse than the HCR's, and worse than ligation alone (74). Taken together, the data clearly suggest that in this “disease prone” predicated on the low intrinsic aerobic capacity phenotype, active exercise is capable of inducing either benefit or harm depending on when it is administered, and the underlying phenotype. In this setting, HCR had about 500% greater intrinsic running capacity than LCR. The impact of active exercise in the HCR was more difficult to evaluate, as the underlying phenotype made it difficult to generate equivalent injury from the hind limb occlusion. At the same time, HCRs were resistant to the occlusion-induced damage, and in contrast to what was seen with cardiac responses to ischemia, the higher aerobic capacity did provide some level of protection. The results are summarized in Table 2.

**Table 2:** The influence of exercise before and after occlusion on vascular recovery, angiogenesis and inflammation in the HCR and LCR phenotypes (74).

**Ex-pre**	**Occlusion**	**Ex-post**	**Phenotype**	**Vascular recovery**	**Angiogenic marker**	**Inflammatory marker**
No	Yes	No	HCR	↑	**–**	**–**
			LCR	↓↓	↑↑	**–**
Yes	Yes	No	HCR	**–**	↓↓	↓
			LCR	↑↑	↑↑	↑
No	Yes	Yes	HCR	↑↑	↓↓	↑↑
			LCR	↓↓	↑	↓↓

*Exercise 2 weeks before ligation (Ex-pre); exercise 2 weeks after ligation (Ex-post); high capacity runner rat (HCR); low capacity runner rat (LCR); Vascular recovery includes morphologic indices such a capillary density and capillary contacts/muscle fiber; Angiogenic markers include gene changes by PCR for targets such as VEGF (vascular endothelial growth factor), VEGF receptors and angiopoietin; Inflammatory markers include gene and protein changes (ELISA) for cytokine markers such as IL-6 (Interleukin 6) and TNF (tumor necrosis factor). Upward arrows indicate increased levels of parameters while downward arrows indicate decreased levels of parameters*.

Our lab was the first to demonstrate that the LCRs are insulin resistant, prone to metabolic syndrome, and showed significantly greater weight gain on high fat diets (85, 86), which has since been established in multiple other studies, contributing to the characterization of the LCRs as “disease prone” (61–64). Obesity and diabetes were not specifically induced in the exercise pre-and post-ligation studies. Still given the established characteristics of the LCR model, our results generally are consistent with those reported by Lyu et al. (75). Our findings indicated that exercise after ligation is beneficial and protected against ischemic injury only in the HCR animals, but was harmful in the LCRs (65). On the other hand, exercise before the ligation seemed beneficial and protected from the ischemic insult in the LCRs, while exercise before the ligation in HCR didn't influence much on the protection against vascular injury. Clearly the relationship between timing of exercise and the presence of co-morbidities may significantly influence the potential for a beneficial outcome with an exercise protocol in animal models of PAD, and humans with PAD.

## Contraindications for exercise

Contraindications for exercise refer to scenarios where physical exercise likely will fail to yield improvement in the quality of life, and where the probability for overt harm is greater than the limited potential for benefit. Although both the lungs and heart benefit greatly from exercise, they tend to suffer when intensive physical activities are performed by patients suffering from pre-existing pulmonary and heart disorders (8). A study by Pederson and Saltin in 2006 showed that exercise is contraindicated in coronary heart disease (CHD) patients until the condition has become stable, after about 5 days (8). Other frequent contraindications to exercise include aortic stenosis, dyspnea at rest, myocarditis, endocarditis, pericarditis, fever and high blood pressure (8). In 1975, Black et al. found that strenuous physical activity can cause injury to coronary plaques, which may cause occlusion of coronary arteries (87). However, recently regular exercise is recommended for patients with coronary artery disease after taking the complete history and examination and putting the patients on a graded exercise test to rule out and treat unstable angina or ischemia before recommending the exercise training in those patients (88).

Contraindications also are common in bone, muscle and joint disorders, such as rheumatoid arthritis and osteoarthritis, when there is acute inflammation or training aggravates pain (8). This discomfort also is commonly associated with patients suffering from cardiopulmonary disorders such as pleuritis and pericarditis (8). Pederson et al. further recommend that osteoporosis patients should only engage in those physical activities associated with minimal risk of falling (8). Other disorders associated with contraindications to exercise include diabetes (type I and II) when there is hyperglycemia (>2.5 g/L) with or without hyperketonuria (3 g/L) (2). The damaging effects of eccentric muscle contractions (89) in metabolic syndrome-related diseases such as dyslipidemia, insulin resistance, and obesity are not contraindicated, *per se*, but the comorbidities may limit the overall exercise capacity (8). Uncontrolled hypertension (systolic > 180 mm Hg, or diastolic > 105) also is a contraindication for exercise unless lowered pharmacologically (90).

## Conclusion

Regular exercise is extremely beneficial in the promotion of health and treatment of diseases. Moreover, findings from various studies have revealed that exercise promotes both the well-being and lifespan of an individual. Hence, researchers now consider exercise as a drug. However, the use of exercise as a therapeutic is relatively unsophisticated, and practically speaking, not held to the same standards as pharmacologically based therapeutics. The presumed “pluripotent” benefit of exercise in virtually every scenario examined regardless of etiology suggests that there is much that is still poorly understood about exercise, and there are circumstances where exercise might be harmful. Prescribing exercise therapy for some disease conditions such as PAD with diabetes requires much better data than currently exists, and some animal studies maybe helpful in designing future clinical trials. While exercise may mitigate the loss of perfusion in some conditions, if a severe decrease in the blood flow to the heart, lung or peripheral extremities already exists, exercise likely will cause harm rather than benefit, and should be avoided until the blood flow can be stabilized/improved. Exercise is a “stress test” of the system, and exercise intolerance is often the very basic hallmark of cardiopulmonary disease.

The current popular trends tend to co-mingle the benefits of exercise as disease prevention, vs. the potential risks of exercise as applied as disease treatment as being equivalent and so far at least, the data clearly indicates that is not the case. With the explosion of genomics, and the strong heritability of exercise traits, it seems logical that exercise will have to follow the same guidelines being developed for other “personalized medicine” approaches before it will realize both its opportunities for benefit, and avoid its opportunities for harm.

## Author contributions

All authors listed have made a substantial, direct and intellectual contribution to the work, and approved it for publication.

### Conflict of interest statement

The authors declare that the research was conducted in the absence of any commercial or financial relationships that could be construed as a potential conflict of interest. The handling editor declared a shared affiliation, though no other collaboration, with one of the authors SB at the time of the review.
